# Voices of Innovation: Reflective Report on Integrating Artificial Intelligence–Simulated Mental Health Patient Scenarios Into Undergraduate Nursing Education in the United Arab Emirates

**DOI:** 10.2196/78161

**Published:** 2026-03-12

**Authors:** Amina Ahmad, Janisha Kavumpurath, Raheesa Kader, Muna Altamimi, Monia El Hajj, Fatma Refaat Ahmed Ahmed, Muhammad Arsyad Subu, Taliaa Yafei, Hind Rashed Ali, Idil Saleh, Nabeel Al-Yateem

**Affiliations:** 1 Department of Nursing, College of Health Sciences, University of Sharjah, University City Road, P.O. Box 27272, Sharjah, University City, United Arab Emirates, 971 605057528 ext 00971605057528; 2 Department of Critical Care and Emergency Nursing, Critical Care & Emergency Nursing, Faculty of Nursing, Alexandria University, Alexandria, Egypt; 3 Faculty of Nursing, Ras al-Khaimah Medical and Health Sciences University, Ras al-Khaimah, United Arab Emirates

**Keywords:** mental health nursing, simulation training, artificial intelligence, AI, nursing education, clinical skills, experiential learning

## Abstract

Limited clinical placements for mental health courses in the United Arab Emirates have made it difficult to provide consistent experiential learning for undergraduate nursing students. As a result, nurse educators are considering technology-enabled learning approaches to deliver clinical skills training. This Viewpoint presents a reflective, theory-informed account of the first-year integration of an artificial intelligence (AI)–enabled, voice-interactive simulated patient into an undergraduate mental health nursing practicum. Grounded in Kolb’s experiential learning cycle and aligned with established simulation best practices, the initiative was designed to support therapeutic communication, psychiatric assessment, and clinical reasoning through structured prebriefing, immersive interaction, and guided debriefing. The paper describes the educational rationale, scenario development, implementation processes, and contextual challenges encountered during real-world deployment across university and clinical environments. AI-supported simulations offered a standardized and psychologically safe context for students to engage with complex psychiatric scenarios, particularly when direct patient interaction is constrained. We discuss operational insights related to technical reliability, environmental requirements, faculty preparation, and assessment integration alongside considerations for scalability and sustainability in resource-limited settings. While AI-supported objective structured clinical examinations have been incorporated to support assessment consistency, formal psychometric validation and outcome comparisons have not been undertaken at this stage. By sharing lessons learned from early implementation, this Viewpoint contributes practical insights for nursing educators facing similar structural constraints. AI-enabled simulation is presented as a strategic complement to, rather than a replacement for, traditional clinical placements, with future empirical research needed to evaluate educational outcomes and long-term impact.

## Introduction and Background

The United Arab Emirates (UAE) admitted its first cohort of bachelor’s-level nursing students in 1998. At that time, enrollment was small, and the limited number of hospitals could accommodate clinical training needs [1]. Since then, rapid population growth to approximately 10 million—largely driven by expatriates—together with major investment in health services has been matched by a sharp expansion in nursing education. More than 15 nursing training institutions are now operating in the country [1]. Although the number of approved clinical training sites has increased, it has not kept pace with the growth in programs [1]. As a result, many institutions have turned to simulation-based training due to competition for clinical placements.

This challenge is not unique to the UAE. Globally, nursing programs continue to face limits in clinical capacity due to workforce shortages, rising student enrollment, and increasing patient acuity. Recent literature also suggests that relying solely on clinical simulation to address these pressures is not sustainable, particularly as both programs and student numbers continue to grow [2-4].

At the same time, simulation technologies have advanced. In addition to low- and medium-fidelity simulators, newer options such as virtual reality (VR) and artificial intelligence (AI)–powered conversational agents have broadened how simulation can be used in nursing education [5,6]. For these technologies to have maximum educational value, however, they should be integrated as a structured learning opportunity rather than treated as an “add-on.” In practice, this means aligning their use with Kolb’s experiential learning cycle: concrete experience, reflective observation, abstract conceptualization, and active experimentation.

Psychiatric nursing education presents additional challenges that are less common in other areas of health care education. Limited psychiatric facilities, complex ethical issues, student vulnerability, and the risk of exposure to sensitive cases reduce opportunities for repeated practice of key interventions [7,8]. Evidence indicates that simulation in psychiatric education can offer a controlled environment where learners can practice communication, emotional regulation, and clinical decision-making skills when real clinical exposure is limited [7,9,10].

These constraints are particularly evident in the UAE, where psychiatric clinical placements are relatively few. Providing every student with high-quality, guided exposure in mental health settings is therefore difficult. To reduce placement pressure while maintaining—and potentially improving—learning quality, we pilot tested a technology-based simulation approach. Specifically, we used high-immersion VR environments combined with AI-based simulated patients to support complex psychiatric interactions at scale.

Early evidence suggests that AI-simulated patients can improve realism, standardization, and scalability, offering psychologically safe opportunities for deliberate practice in communication and clinical reasoning [11,12]. During periods of system strain, such as the COVID-19 pandemic, AI applications aligned with Industry 4.0 have also been used to support health care work processes by strengthening service delivery infrastructure rather than replacing professional judgment [13,14].

This paper describes our first-year experience embedding an AI voice interaction system—designed to simulate a patient with a mental disorder—into a mental health nursing practicum course. Using a reflective approach grounded in relevant theory, we examine the implementation processes and challenges associated with integrating AI simulation technologies into mental health nursing education, particularly within structural limits related to clinical placement capacity.

## The Indispensable Role of Clinical Training

Practical experience is the foundation of nursing education. It allows students to translate theory into patient care while building psychomotor, cognitive, and interpersonal skills and strengthening professional identity, confidence, and competence [15,16]. However, deliberate practice is often limited by institutional inefficiencies, poorly aligned programs, and insufficient supervision. These constraints can reduce student satisfaction and slow skills development [17,18].

These pressures are most pronounced in mental health nursing. With only 3 dedicated psychiatric facilities across the country, students may face stigma, inconsistent staff attitudes, and high stress, which can contribute to anxiety or even avoidance of the specialty despite the need for well-prepared practitioners [19,20]. Simulation can help bridge this gap by offering a controlled environment—using manikins and standardized patients—where learners can practice safely, reduce anxiety, and build competence. However, traditional simulation is often resource intensive [21,22].

Newer immersive approaches may extend practice opportunities further. VR can simulate complex and dynamic psychiatric scenarios, while AI-powered chatbots can support adaptive, realistic therapeutic conversations. Together, these tools can expand access to intentional practice and reflection, particularly where clinical exposure is limited [23-25].

## The UAE Context

In the UAE, particularly in the Northern Emirates, the number of inpatient psychiatric beds has traditionally been limited. Prior to 2020, nursing students were placed on rotation in only 2 specialist psychiatric hospitals—both of which already shared a limited number of spots with medical and allied health programs. Just as the demand for these placements became acute in the aftermath of the COVID-19 pandemic—due to a marked rise in postpandemic nursing enrollments [25]—the number of available placements plummeted. At the University of Sharjah, only 35% of students enrolled in the 2023‐2024 practicum in mental health nursing were able to secure a traditional placement.

Outpatient psychiatric clinics offered little relief. As some of these clinics are part of for-profit multispecialty hospitals, patients and families often refuse to cooperate with student interviews to avoid the stigma associated with being labeled “psychiatric.” This has put student cohorts at risk of not meeting course objectives related to conducting mental status examinations (MSEs) or practicing de-escalation skills—skills they may only perform occasionally, if at all.

The combination of (1) a chronic shortage of psychiatric training facilities, (2) the diversion of such facilities during the pandemic, and (3) an increasing number of students has created a pressing need for an effective and scalable simulation option. Fortunately, the current state of VR simulation and AI-driven simulated patients has reached a level of maturity that allows for the realistic replication of complex psychiatric scenarios.

This reflection, therefore, assesses the introduction of an AI-based VR simulation program within an undergraduate mental health nursing practicum course. The aim is to evaluate whether immersive, intelligent simulation can bridge the clinical exposure gap, ensure that students meet identified competencies, and establish a sustainable framework for teaching mental health nursing in developing contexts such as the UAE.

## The Experience: Development of the AI-Driven Solution

Simulation has proven to be of enormous benefit to the nursing industry. The maturity and rapid evolution of AI technologies, combined with the severe shortage of mental health training placements, were the primary drivers behind the introduction of AI-driven simulated patients that can interact with students through voice.

Prior to implementation, an intensive preimplementation workshop was conducted over 1 week with all participants. This workshop reinforced core concepts in mental health care, including models of therapeutic communication, MSE principles, patient education strategies, risk assessment within MSEs, and the application of the nursing process to mental health scenarios. In parallel, participants attended theoretical classes twice a week focused on mental health nursing concepts. This convergence of activities during the preimplementation phase ensured that students were adequately equipped with foundational knowledge, thereby supporting improved critical thinking and readiness to engage with AI-based simulated patient interactions.

The next step involved the creation of realistic psychiatric patient scenarios. Using diagnoses and symptoms identified in the *Diagnostic and Statistical Manual of Mental Disorders, Fifth Edition, Text Revision* (*DSM-5-TR*) reference guide [26], scenarios were developed to reflect common mental health conditions such as schizophrenia, bipolar I disorder, and major depressive disorder with psychotic features.

Each patient scenario included the following elements: (1) in-depth information about the patient’s history, (2) detailed accounts of past mental and emotional circumstances, and (3) clinical information, including pharmacological management.

An example of the schizophrenia patient simulation is presented in Textbox 1.

To enhance content validity, these scenarios were developed in collaboration with mental health faculty members and university professors with practical experience as psychiatric mental health nurses.

Textbox 1. **Simulation scenario.**

Age and gender: 24-year-old female

Presenting concern: “They’re watching me through the ceiling vents. I can hear them talking about me.”

Background

Ms. L, a 24-year-old university student, was brought to the emergency department by her mother following several weeks of increasingly unusual behavior. Her mother reports that Ms. L has become socially withdrawn, staying isolated in her room, skipping university classes, and speaking less. In the last two weeks, she began covering her bedroom vents with tape and voiced fears that people were monitoring her. She refuses food prepared by family, citing contamination concerns. She is occasionally heard speaking or laughing when alone. During the assessment, she avoids eye contact, speaks slowly, and appears distracted by internal stimuli. She denies suicidal ideation but displays significant mistrust. She has no prior psychiatric history or substance use, though a maternal uncle was diagnosed with schizophrenia.

Medications

- Olanzapine 10 mg at bedtime (initiated)
- Lorazepam 1 mg (as needed) for agitation
- (Note: initially resistant to treatment; currently partially adherent under supervision)

Nonverbal cues

- Frequently looks around the room suspiciously
- Avoids direct eye contact
- Flat affect
- Occasionally murmurs to herself
- Limited verbal output

Assessment and learning objectives

- Apply therapeutic communication techniques to establish rapport
- Conduct a comprehensive mental status examination
- Assess safety risks and patient needs based on behavioral cues
- Provide psychoeducation on medication adherence and symptom management
- Identify the *Diagnostic and Statistical Manual of Mental Disorders, Fifth Edition, Text Revision*, diagnostic features of schizophrenia based on the patient scenario

A set of simulation prompts was also designed to promote authentic responses during student interactions. Each prompt instructed the AI to fully embody the psychiatric patient’s persona, displaying appropriate emotional expressions, speech patterns, and symptoms consistent with the diagnosed condition. Furthermore, the prompts included descriptions of nonverbal behaviors—such as sighing, looking away, leaning forward, or avoiding eye contact—prior to verbal responses. Accurately interpreting nonverbal cues is a critical component of therapeutic communication.

The AI simulation prompt is illustrated in Textbox 2.

Textbox 2. **Standardized simulation prompt.**

“You are going to role-play a psychiatric patient for a clinical nursing simulation. I will give you a case scenario, and you must respond strictly in character as the patient described.

Use realistic voice tone, emotion, and language that reflect the symptoms and emotional state of the patient.

Begin by giving a brief behavioral description before each verbal response—for example: ‘sighs and looks away before speaking’ or ‘fidgets with hands, then answers softly’.

These nonverbal behaviors are important for the student to assess. Do not speak unless the student initiates the conversation.

Respond naturally to the student’s questions, but remain within the emotional and behavioral context of the case.

You must not break character. Speak only in English.”

## Implementation and Integration Into Clinical Education

Students were scheduled for structured AI simulation activities as part of their clinical practicum hours. They were provided with minimal background information prior to participation—mirroring actual psychiatric practice, where building rapport and using effective communication strategies are essential to obtain a comprehensive patient history.

Before each structured simulation activity, a prebriefing session was conducted to orient students to key aspects related to learning objectives, psychological safety, and appropriate communication strategies, particularly in relation to risk and professionalism. During the simulation, students engaged in conversations with AI-simulated patients to conduct comprehensive MSEs; deliver psychoeducation on medication adherence; and perform risk assessments for suicidal ideation, self-harm, and aggression. On the basis of their assessments, students developed individualized patient care plans.

Faculty members from the school observed these simulation activities either live or via recordings. Each simulation was followed by a debriefing session, which facilitated reflective learning and enabled students to critically evaluate their clinical decision-making processes and develop more effective communication strategies.

While the initial plan was to conduct these AI simulations solely at the university, a need arose to integrate them into students’ hospital rotations. In outpatient settings, particularly within private hospitals, students often encountered barriers to direct patient interaction. The stigma surrounding mental illness led some patients to decline student participation in interviews, even though these hospitals were not strictly mental health facilities. Many patients also sought services discreetly, further limiting opportunities for students to participate in consultations. These factors posed challenges to the development of students’ therapeutic communication skills.

To address these issues and maintain continuity in learning, AI simulations began to be used within hospital environments. However, logistical challenges emerged. Unreliable Wi-Fi connections often disrupted simulation sessions, leading to delays or misinterpretations by the AI tool. Finding suitable, private locations for simulation within hospitals was also difficult, and background noise occasionally interfered with the AI’s speech recognition capabilities.

Despite these operational challenges, the implementation of AI simulations in medical training remained largely effective. Students gained valuable experience in therapeutic communication, psychiatric assessment, risk evaluation, and critical thinking—skills they might not have been able to develop otherwise.

## Extension Into Assessment: AI-Supported Objective Structured Clinical Examinations

On the basis of the success achieved through AI-powered simulated patient interactions during clinical training, the simulated activity was incorporated into the assessment framework through the use of objective structured clinical examinations (OSCEs). To ensure a fair platform for all examinees, students participated in a set of stations designed to assess various skills in the domain of psychiatric nursing competence.

Each participant was randomly assigned a case scenario from a pool of prepared cases. Each scenario contained core clinical information typically found in a hospital patient file, including the presenting problem, relevant history, psychiatric diagnosis, current medications, and risk factors. Students were given 5 minutes of preparation time before beginning the clinical activity based on the case.

The first station assessed therapeutic communication skills. Students engaged in a live voice interaction exercise with the AI-simulated patient, applying key concepts such as empathy, active listening, validation, and rapport building. Performance was evaluated using a structured rubric.

The second station was a viva voce session in which students’ applied knowledge of the case was evaluated. They were asked questions to assess their understanding of the clinical presentation, differential diagnosis, psychopharmacological approach, and evidence-based nursing management.

The third station involved the demonstration of a full MSE. Students conducted a guided MSE on the AI-simulated patient through active engagement in a voice-only conversation. Although the interaction was audio based, the prompts were designed to include brief behavioral cues (eg, “fidgets with hands” and “avoids eye contact”) preceding verbal responses. This allowed students to evaluate domains such as appearance, behavior, speech, mood, affect, thought process, cognition, insight, and judgment—drawing on both content and contextual cues. This setup encouraged the use of clinical judgment under realistic conditions.

Following the simulation stations, students were given 30 minutes to record their assessment findings and develop a corresponding nursing care plan. This component measured their ability to synthesize information from the simulation and formulate an appropriate care plan.

**Figure 1.**
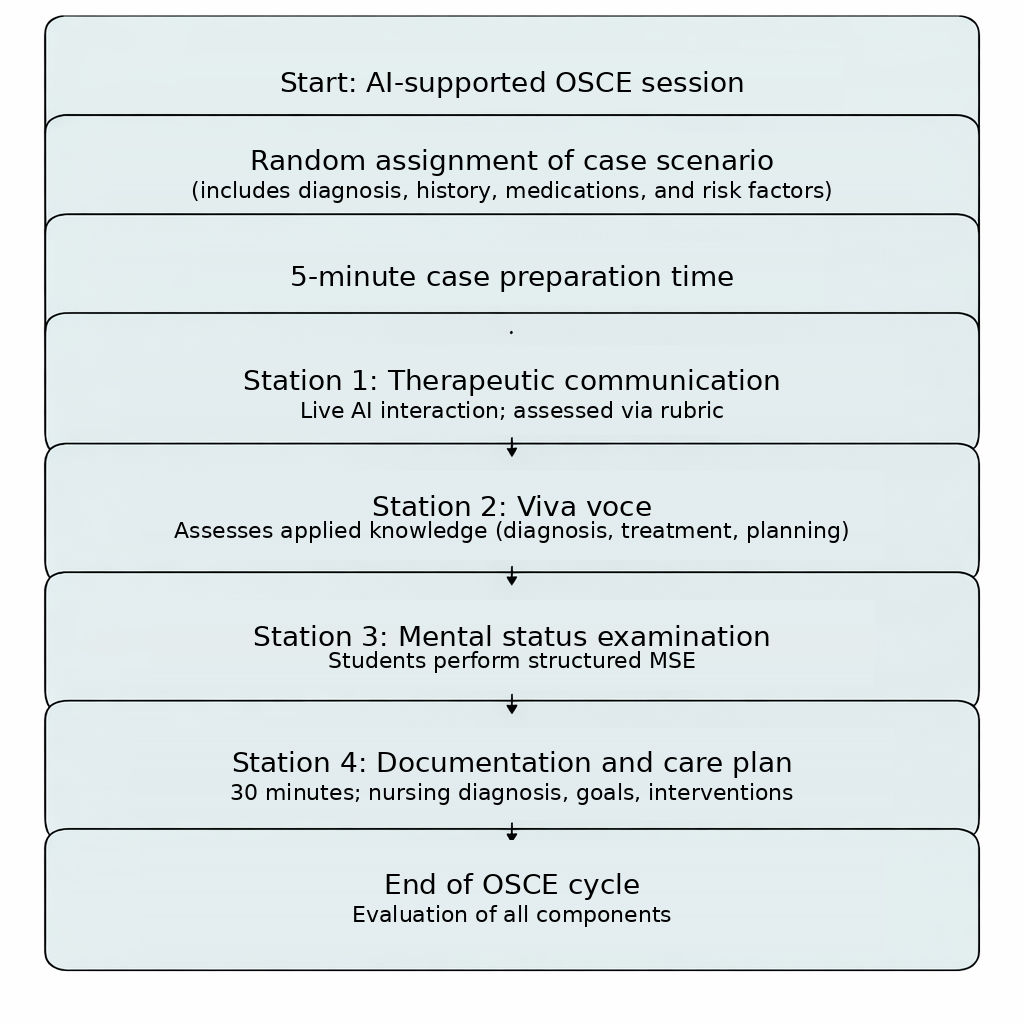
Flowchart of artificial intelligence (AI)–integrated objective structured clinical examinations (OSCE) structure in mental health nursing education. MSE: mental status examination.

The inclusion of AI-led simulated patients throughout the OSCE ensured consistency in the assessment process and eliminated variability commonly associated with live patients or inconsistencies in standardized patient performances. The structural layout of the OSCE is illustrated in Figure 1.

Although the AI-supported OSCEs were intended to enhance standardization and authenticity in student assessment, the current implementation did not include formal validation, reliability testing, or outcome comparisons. These factors should be addressed in future studies.

## Opportunities Created by AI Integration

The integration of AI simulation provided a range of learning opportunities that extended beyond simply addressing the clinical placement gap. From an instructional design perspective, the simulation offered a balanced opportunity for students to engage with complex mental health scenarios—particularly important in contexts where hospital placements are dependent on availability. It allowed for consistent exposure to essential mental health nursing skills.

The model also introduced flexibility in curriculum delivery. The use of AI simulations enabled the incremental development and deployment of patient scenarios, allowing faculty to adapt to changes in enrollment without placing additional strain on clinical partners. This is particularly valuable in environments where expanding clinical capacity is not feasible, even as student numbers increase.

Another key learning affordance was the provision of immediate feedback. Faculty members were able to observe students during simulation activities and conduct debriefing sessions focused on clinical decision-making, communication strategies, and the application of professional judgment.

Importantly, the simulations fostered an environment of psychological safety in which students could develop their therapeutic communication and psychiatric assessment skills. Some students reported feeling anxious about interacting with clients with mental illness during their initial clinical exposures. Practicing these interactions in a controlled, simulated setting helped them build familiarity and confidence.

Beyond mental health education, this strategy shows potential for broader application across other areas of nursing education, including physical assessment and adult medical-surgical nursing. Incorporating AI-enabled voice interaction into existing simulation systems could further enhance the learning experience.

These observations highlight the educational benefits of AI simulation but do not evaluate its overall effectiveness, underscoring the need for future research and formal evaluation.

## Challenges and Lessons Learned

Although the AI-based simulation model provided valuable learning opportunities, several challenges emerged during its application, many of which informed improvements in later versions.

*Reliability* was identified as a fundamental requirement. Stable Wi-Fi connectivity was essential for uninterrupted speech communication; however, occasional network failures occurred. These disruptions, particularly during periods of high use, suggested the potential need for multiple subscription services to ensure consistent access.

*Environmental factors* also influenced the quality of the simulations. Conducting sessions in sound-absorbing environments proved critical, as background noise in real-world settings sometimes interfered with speech recognition, leading to inconsistent or inaccurate responses. This highlights the importance of designated simulation spaces to maintain high-quality interactions.

*Limitations in nonverbal communication* were another noted issue. While verbal communication was effectively simulated, the lack of dynamic facial expressions and gestures reduced the realism of certain interactions. Although prompts included descriptions of nonverbal cues before verbal responses, future enhancements could incorporate AI avatars or graphical interfaces to improve the simulation of nonverbal behaviors.

*Faculty readiness* was also a key consideration. Successful facilitation required educators not only to be proficient with the technology but also to manage essential instructional tasks, such as conducting prebriefs, monitoring learner engagement, leading debriefings, and aligning the simulation with learning outcomes.

In light of these challenges and limitations, AI-enhanced simulation should be viewed as a *complementary strategy* in clinical training rather than a substitute for traditional clinical experiences. Its value lies in augmenting, not replacing, real-world learning.

## Reflection and Future Directions

The inclusion of ChatGPT Voice in the Mental Health Nursing Practicum course at the University of Sharjah was a major milestone in applying innovation to nursing education. The innovation sought to fill the gap that had been experienced in psychiatric clinical placements by applying AI to design a dynamic virtual environment to better link theory and practice.

Looking ahead, there are a number of opportunities that can be identified to further improve the educational quality of such a simulation-based system. Increasing the number of clinical simulations available that relate to a broader set of psychiatric disorders can potentially create a more inclusive learning environment. The addition of algorithms related to theories of emotional intelligence can also increase the realism of emotionally based patient reactions, thereby enhancing the level of therapeutic involvement that can take place during a student-patient interaction.

Future studies might also examine student satisfaction and learning results using AI-supported simulation activities. These studies would offer significant insights into the efficacy and continuing improvement of such educational instruments.

Notably, the new approach aligns with the international standards that promote the convergence of technology and creativity in medical education programs [27,28]. Furthermore, the approach aligns with the UAE Vision 2021 and Centennial 2071 strategies in innovation and the convergence of AI in health-related education programs in the UAE [29]. In fact, it aligns with the Strategic Plan 2024‐2030 of the University of Sharjah [30].

## Conclusions

With unprecedented challenges experienced in sourcing clinical placement opportunities for nurses, the ChatGPT Voice Interaction simulation embodies the potential for AI simulation to offer a sustainable approach to mental health nurse education.

Through its structured implementation based on theories, the approach enabled opportunities for the engagement of skills in therapeutic communication, psychiatric assessment, and planning for a safe and fair learning context.

This project shows how new technology could be conceptualized to work well with existing approaches to education, especially where access to clinical training facilities is limited. The developing nature of technology means that well-structured uptake of AI simulation learning could help to ensure education continuity by preparing nursing graduates to handle the growing complexity of modern-day health care.

## Abbreviations

AI: artificial intelligence
*DSM-5-TR*: *Diagnostic and Statistical Manual of Mental Disorders, Fifth Edition, Text Revision*
MSE: mental status examination
OSCE: objective structured clinical examination
UAE: United Arab Emirates
VR: virtual reality
